# Co-circulation of *Plasmodium vivax* and Arboviruses in the Brazil-French Guiana Border

**DOI:** 10.1590/0037-8682-0226-2025

**Published:** 2026-02-13

**Authors:** Marcelo Cerilo-Filho, Estela dos Santos Medeiros, Marcelo de Lima Arouca, Marrara Pereira Sampaio, Rubens Alex de Oliveira Menezes, Margarete do Socorro Mendonça Gomes, José Rodrigo Santos Silva, Andréa Regina de Souza Baptista, Luciane Moreno Storti-Melo, Ricardo Luiz Dantas Machado

**Affiliations:** 1 Universidade Federal Fluminense, Instituto Biomédico, Centro de Investigação de Microrganismos, Niterói, RJ, Brasil.; 2 Universidade Federal Fluminense, Programa de Pós-Graduação Stricto Sensu em Microbiologia e Parasitologia Aplicadas, Niterói, RJ, Brasil.; 3 Universidade Federal de Sergipe, Programa de Pós-Graduação Stricto Sensu em Biologia dos Agentes Infecciosos e Parasitários, São Cristóvão, SE, Brasil.; 4 Universidade Federal Fluminense, Programa de Pós-Graduação Stricto Sensu em Ciência e Biotecnologia, Niterói, RJ, Brasil.; 5 Universidade Federal do Amapá, Programa de Pós-Graduação Stricto Sensu em Ciências da Saúde, Macapá, AP, Brasil.; 6 Superintendência de Vigilância em Saúde do Estado do Amapá, Macapá, AP, Brasil.

**Keywords:** Arbovirus infection, Malaria, Mixed infection, Public health, Vector borne diseases

## Abstract

**Background:**

Co-circulation of *Plasmodium vivax* and arboviruses is a significant but underexplored public health concern in tropical regions.

**Methods:**

We analyzed 300 serum samples collected between 2014 and 2015 from the Brazil-French Guiana border, including 102 malaria cases and 198 controls, using rapid tests for Immunoglobulin M (IgM), Immunoglobulin G (IgG), and Non-Structural Protein 1 (NS1).

**Results:**

High IgG reactivity for dengue and chikungunya viruses was observed. Co-reactivity was frequent. Zika virus detection was minimal.

**Conclusions:**

The overlapping transmission of *P. vivax* and arboviruses highlights the need for integrated surveillance strategies to better address co-circulation/co-infection risks.

Malaria caused by *Plasmodium vivax* and arboviral infections, such as Dengue virus (DENV), Chikungunya virus (CHIKV), and Zika virus (ZIKV), coexist in many tropical regions, particularly in the Amazon rainforest, where environmental conditions favor their transmission. Overlapping symptoms, including fever, headache, and myalgia complicate clinical diagnosis and hinder timely management^1^ ^,^ ^2^. In this region, *P. vivax* malaria remains highly prevalent, whereas arboviruses circulate simultaneously in both the urban, peri-urban and rural areas^3^.

Previous studies from our group demonstrated that co-infection with *P. vivax* and intestinal parasites was associated with a Th2-skewed immune response^4^ ^,^ ^5^, and that co-infection with *P. vivax* and Human Parvovirus B19 increased parasitemia and prolonged gametocytemia^6^. These observations highlighted the importance of identifying additional pathogens that may influence the clinical course of *P. vivax* malaria. A global systematic review conducted by our group showed that co-infections involving *P. viv*ax and arboviruses, particularly *P. vivax* + DENV and *P. vivax* + CHIKV, may intensify clinical manifestations^7^.

However, evidence regarding the co-circulation of arboviruses in regions endemic for *P. vivax* malaria remains scarce, including along the Brazil-French Guiana border, an area of intense population movement and ecological overlap between *Anopheles* and *Aedes* vectors. Therefore, the objective of this study was to investigate the co-circulation of DENV, CHIKV, and ZIKV among febrile individuals seeking *P. vivax* malaria diagnosis in Oiapoque, Amapá, Brazil-French Guiana border. Accordingly, we conducted a cross-sectional study with a subset of 102 *P. vivax*-positive and 198 *P. vivax*-negative samples that were previously analyzed elsewhere^8^. Samples were collected between November 2014 and November 2015 from the municipality of Oiapoque, located in the state of Amapá, Brazil on the French Guiana border. All sample collection was conducted at the official Malaria Care Units, which are the primary points of care for individuals presenting with fever in this region. These facilities play a central role in malaria diagnosis and treatment and, therefore, represent a strategic setting for investigating the concurrent circulation of other infectious agents that share similar clinical manifestations. All samples were stored at -80 °C at the Centro de Investigação de Microrganismos in the Universidade Federal Fluminense (Niterói, Rio de Janeiro, Brazil) until use. 

The target population consisted of individuals who sought care for fever and underwent routine malaria testing. All participants were evaluated by a trained nurse who used a standardized clinical form to record their signs and symptoms. Eligibility required patients to report the onset of fever within the previous 72 h, allowing the study to capture acute episodes more reliably. After clinical screening, all participants underwent blood collection for malaria diagnosis. This ensured that both *P. vivax*-positive and *P. vivax*-negative individuals were subjected to identical diagnostic procedures, thereby maintaining internal comparability between the groups.

Participants were eligible if they were older than seven years, had lived in Oiapoque for at least five years, and provided written informed consent. The exclusion criteria included pregnancy, comorbidities, and malaria caused by species other than *P. vivax* or mixed-species infections. These exclusions were implemented to reduce the potential confounding factors related to immunosuppression, pregnancy-related physiological changes, and multiple simultaneous infections, which could complicate the interpretation of the results.

*Plasmodium vivax* diagnosis was initially performed using thick blood smear microscopy and subsequently confirmed by nested PCR^8^. DNA extraction was performed using the Wizard® Genomic DNA Purification Kit (Promega Corporation, Madison, WI, USA), according to the manufacturer’s instructions. A combination of parasitological and molecular methods was used to ensure high diagnostic sensitivity and specificity, minimizing the likelihood of false-negative results in the control group.

Arboviral testing was performed using ECO F rapid serological tests (ECO Diagnostic, SD Biosensor, Brazil) on serological samples processed from whole blood collected at the time of care. Both IgM and IgG antibodies were measured for DENV and CHIKV, allowing a distinction between recent and past exposure. For ZIKV, only the IgM assay is available in commercial kits, enabling the detection of acute infection but not prior exposure. In addition, the DENV NS1 antigen test was performed to detect active dengue infection. All tests were performed using an ECO Reader F100 device to ensure standardized interpretation. The procedures were performed in accordance with the manufacturer’s instructions.

Clinical data, including the presence of chills, headaches, myalgia, nausea, vomiting, and body temperature, were collected. These data allowed the characterization of the clinical profiles of participants with and without *P. vivax* malaria, and the assessment of symptom patterns associated with possible arboviral exposure. Additional sociodemographic variables, such as age, sex, and length of residence in the region, were also recorded, as they represent relevant indicators of historical exposure to local vectors.

All samples included in this study were collected with the approval of the Research Ethics Committee of the Universidade Federal do Amapá (Macapá, Amapá, Brazil), under CAAE: 40790120.7.0000.0003.

Among the 300 participants, 102 (34%) were diagnosed with uncomplicated *P. vivax* malaria, whereas 198 (66%) tested negative for *Plasmodium* at the time of collection and were included as the control group. None of the participants in either group required hospitalization. In both groups, the majority of the individuals were male, with a mean age ranging from 28 to 29 years. The participants had lived in Oiapoque for an average of 19-29 years and reported a history of approximately four malaria episodes. All the participants in both groups presented with fever. Symptoms such as chills, headache, myalgia, nausea, and vomiting were more frequent in the malarial group, whereas hemoglobin levels ranged from 7.7-16 g/dL in the malarial group and from 10.6-16 g/dL in the control group. In the malaria group, the mean parasite density was 572.96 parasites/µL **(** Table 1 **).**

**TABLE 1:** Sociodemographic and clinical data of the population included in this study.

Variables	Malarial group (N=102)	Control group (N=198)
Male^a^	73% (N=74)	60% (N=119)
Female^a^	27% (N=28)	40% (N=79)
Age (years)^b^	29 (7-62)	28 (11-74)
Residence time in Oiapoque (years) ^b^	29 (12-79)	19 (10-60)
Number of previous malaria episodes ^b^	4 (2-10)	4 (1-16)
Clinical aspects		
Chills ^a^	34% (N=35)	3% (N=6)
Fever ^a^	100% (N=102)	100% (N=198)
Headache ^a^	78% (N=80)	9% (N=18)
Myalgia ^a^	65% (N=66)	8% (N=16)
Nausea and vomiting ^a^	31% (N=32)	5% (N=10)
Hemoglobin (g/dL) ^b^	13.6 (7.7-16)	13 (10.6-16)
Parasite density (parasites/µL) ^b^	572.96 (60-30.000)	-

^a^ Percent (total number); ^b^ Mean (range).

Serological testing for arboviruses was conducted on all samples included in this study. A higher frequency of IgG positivity for DENV and CHIKV was observed among individuals not infected with *P. vivax*. Regarding IgM detection, only three individuals tested positive for CHIKV, all of whom were in the *P. vivax*-infected group, thereby suggesting the possibility of a coinfection with both pathogens. In the control group, participants with reactive IgM against DENV, CHIKV, and ZIKV were identified. Notably, only one participant in the control group tested positive for ZIKV IgM. Some participants tested positive for IgG against both viruses (DENV + CHIKV) in both the malaria and control groups **(** Table 2 **)**.

**TABLE 2:** Positivity of IgM, IgG, and NS1 for DENV, CHIKV, ZIKV in samples *P. vivax*-positive and control.

Groups	DENV			CHIKV		ZIKV	DENV+CHIKV	
	IgM	IgG	NS1	IgM	IgG	IgM	IgM	IgG
**Malaria (n=102)**	NR	80 (78.4%)	NR	3 (2.9%)	43 (42.1%)	NR	NR	39 (38.2%)
**Control (n=198)**	4 (2%)	154 (77.8%)	8 (4%)	5 (2.5%)	104 (52.5%)	1 (0.5%)	NR	70 (36.5%)

**NR:** Non-reactive.

Among participants in the control group, eight tested positive for NS1-DENV **(** Table 2 **)**. All of these individuals were also IgM-positive for DENV, suggesting that the detection of the NS1 antigen was associated with an immune response consistent with a recent or acute-phase infection. The simultaneous presence of NS1 and IgM aligns with the immunological window described for DENV, in which the NS1 antigen is detectable mainly during the first few days of viremia, whereas IgM emerges during the early immune response phase^1^ ^,^ ^2^.

The co-circulation and co-infection of *Plasmodium vivax* and arboviral infections represent a significant public health concern, underscoring the complexity of the ecological and epidemiological interactions that facilitate their transmission. Malaria is primarily transmitted by mosquitoes of the *Anopheles*, whereas arboviruses, such as DENV, CHIKV, and ZIKV are vectored by *Aedes* mosquitoes. These vector-borne diseases frequently coexist in tropical and subtropical regions, particularly where environmental conditions favor the proliferation and overlap of both mosquito species^1^ ^-^ ^4^. Factors such as high temperatures, abundant rainfall, and stagnant water bodies contribute to the breeding, survival, and simultaneous presence of these vectors in shared ecosystems^9^ ^,^ ^10^.

The Amazon rainforest provides an extensive ecological niche that supports the coexistence of multiple vector species. Notably, a growing trend has been observed in the urban transmission of arboviral diseases, whereas malaria remains a predominantly rural disease. This divergence is primarily attributed to the greater vector competence of *Aedes*, which reproduce more efficiently in urban environments than *Anopheles* ^2^ ^,^ ^3^ ^,^ ^9^ ^,^ ^10^. Nevertheless, co-infections have become an emerging reality^11^ ^,^ ^12^, facilitated by the frequent movement of individuals between rural and urban settings.

To eliminate disease vectors, the Brazilian Ministry of Health has implemented a national program to reduce mosquito populations. However, the effectiveness of these initiatives may be compromised by strategies that fail to account for species diversity or specific ecological characteristics. For instance, control measures that are effective against *Aedes* spp. may not yield similar results against *Anopheles* spp., and vice versa^1^ ^-^ ^4^. Moreover, the simultaneous circulation of multiple vector species imposes an additional burden on healthcare systems, as the overlapping clinical manifestations of these infections complicate accurate diagnosis and timely treatment. In the Brazilian Amazon, where malaria and arboviruses remain the most prevalent vector-borne diseases^7^ ^,^ ^11^ ^,^ ^12^, such co-infection scenarios are likely to be underreported. 

Our study revealed that a high proportion of individuals tested positive for *P. vivax* and showed serological evidence of prior arboviral exposure (IgG-positive), indicating substantial co-circulation. Additionally, three *P. vivax*-positive individuals were IgM-positive for CHIKV, suggesting probable active coinfection.

Monoinfections with DENV (83.8%), CHIKV (55%), or ZIKV (0.5%) were identified based on IgM and/or IgG seropositivity. The low frequency of ZIKV detection may be attributed to two main factors, including the limitations of this study: (1) the unavailability of a commercial IgG assay for ZIKV from the manufacturer and (2) ZIKV is known to circulate in Brazil, and its distribution remains less extensive than that of DENV and CHIKV. In this context, coinfection with malaria and arboviral diseases cannot be ruled out and should be considered in patients residing in or returning from areas where both types of infections are endemic.

Despite the low number of individuals testing positive for IgM antibodies, cross-reactivity between the antigens of genetically related viruses, such as ZIKV and DENV, may occur, potentially leading to false-positive or false-negative serological results. Accordingly, we acknowledge this as a limitation. However, our findings remain informative within the diagnostic context of this region. Future studies incorporating molecular testing are important to further strengthen and refine these results. Notably, molecular detection of arboviruses can be affected by the time elapsed between sample collection and testing. Since the serological samples analyzed in this study were collected approximately 10 years ago, this represents an additional limitation that may have hindered the recovery of viral genetic material.

Although malaria is caused by protozoans rather than viruses, it can elicit a nonspecific immune response that may induce the production of antibodies capable of interfering with serological assays, including those for arboviruses. This immunological overlap may contribute to false-positive findings in serological testing.

This study identified the co-circulation of *P. vivax* malaria and three arboviruses (DENV, CHIKV, and ZIKV) in the Brazilian Amazon region. However, a notable gap exists in the literature regarding the co-circulation of *P. vivax* with other arboviruses, such as Mayaro and Oropouche, which are also endemic to this region. Therefore, investigating these additional pathogens is essential to better understand the potential for coinfection in malaria-endemic areas. This study was conducted between 2014 and 2015, before the approval of DENV and CHIKV vaccines in 2022 and 2023, respectively^13^ ^-^ ^15^. Future research should incorporate both pre- and post-vaccination data to assess the potential impact of immunization on the dynamics of arboviruses-*Plasmodium vivax* co-circulation and co-infection in endemic settings.

All study participants presented with fever, and some exhibited additional symptoms but tested negative for *Plasmodium*. In these cases, the health unit recommended the use of medications to alleviate symptoms and advised patients to return later for reassessment, if necessary. However, testing for arboviral infections was rarely recommended.

The confirmed co-circulation of three arboviruses (DENV, CHIKV, and ZIKV) and *P. vivax* along the Brazil-French Guiana border underscores the urgent need to strengthen epidemiological surveillance in this region. This co-endemic context poses substantial challenges to public health, as overlapping clinical manifestations and complex pathogen interactions can hinder accurate diagnosis and timely intervention. Investigating patterns of co-circulation and co-infection is therefore critical, not only to improve diagnostic accuracy and inform clinical decision-making, but also to support the development of integrated strategies for the surveillance and control of febrile illnesses in endemic settings.

## Data Availability

The authors confirm that all the data underlying the findings are fully available without restrictions.
